# Rapamycin inhibits hepatitis B virus covalently closed circular DNA transcription by enhancing the ubiquitination of HBx

**DOI:** 10.3389/fmicb.2022.850087

**Published:** 2022-08-11

**Authors:** Yuan Zhang, Liang Li, Sheng-Tao Cheng, Yi-Ping Qin, Xin He, Fan Li, Dai-Qing Wu, Fang Ren, Hai-Bo Yu, Jing Liu, Juan Chen, Ji-Hua Ren, Zhen-Zhen Zhang

**Affiliations:** ^1^The Key Laboratory of Molecular Biology of Infectious Diseases Designated by the Chinese Ministry of Education, Chongqing Medical University, Chongqing, China; ^2^Department of Infectious Disease, Children’s Hospital of Chongqing Medical University, Chongqing, China; ^3^National Clinical Research Center for Child Health and Disorders, Ministry of Education Key Laboratory of Child Development and Disorders, Chongqing, China; ^4^Chongqing Key Laboratory of Child Infection and Immunity, Chongqing, China; ^5^Department of Gastroenterology, Chongqing University Three Gorges Hospital, Chongqing, China; ^6^Department of Endocrine and Breast Surgery, The First Affiliated Hospital of Chongqing Medical University, Chongqing, China; ^7^Chongqing Key Laboratory of Sichuan-Chongqing Co-construction for Diagnosis and Treatment of Infectious Diseases Integrated Traditional Chinese and Western Medicine, Chongqing, China; ^8^Department of Laboratory Medicine, Chongqing Hospital of Traditional Chinese Medicine, Chongqing, China

**Keywords:** rapamycin, hepatitis B virus, HBx, covalently closed circular DNA, transcription

## Abstract

Hepatitis B virus (HBV) infection is still a serious public health problem worldwide. Antiviral therapies such as interferon and nucleos(t)ide analogs efficiently control HBV replication, but they cannot eradicate chronic hepatitis B (CHB) because of their incapacity to eliminate endocellular covalently closed circular DNA (cccDNA). Thus, there is a necessity to develop new strategies for targeting cccDNA. As cccDNA is difficult to clear, transcriptional silencing of cccDNA is a possible effective strategy. HBx plays a vitally important role in maintaining the transcriptional activity of cccDNA and it could be a target for blocking the transcription of cccDNA. To screen new drugs that may contribute to antiviral therapy, the ability of 2,000 small-molecule compounds to inhibit HBx was examined by the HiBiT lytic detection system. We found that the macrolide compound rapamycin, which is clinically used to prevent acute rejection after organ transplantation, could significantly reduce HBx protein expression. Mechanistic studies demonstrated that rapamycin decreased the stability of the HBx protein by promoting its degradation *via* the ubiquitin-proteasome system. Moreover, rapamycin inhibited HBV RNA, HBV DNA, and cccDNA transcription levels in HBV-infected cells. In addition, HBx deficiency abrogated the inhibition of cccDNA transcription induced by rapamycin. Similar results were also confirmed in a recombinant cccDNA mouse model. In summary, we report a new small-molecule, rapamycin, which targets HBx to block HBV cccDNA transcription and inhibit HBV replication. This approach can identify new strategies to cure CHB.

## Introduction

Chronic hepatitis B (CHB) is one of the main causes of liver cirrhosis and hepatocellular carcinoma (HCC) (Hu et al., 2019; Revill et al., 2019). According to the WHO, there were around 257 million CHB virus carriers, and 887,000 deaths from Hepatitis B virus (HBV)-related complications in 2015 (World Health Organization [WHO], 2017).

During the process of HBV replication, the 3.2-kb genome of HBV can form covalently closed circular DNA (cccDNA) in the nucleus, which is difficult to eliminate (Gish et al., 2015). As the template for all HBV RNAs transcription, cccDNA plays a crucial role in the HBV life cycle and accounts for HBV chronicity (Hong et al., 2017). Despite the strong inhibition applied by interferon (IFN) and nucleos(t)ide analogs (NAs) on HBV DNA, they cannot clear cccDNA and resolve the persistence of HBV infection (Lok et al., 2016). Therefore, the identification of new drugs targeting cccDNA is urgently needed. Since it is unlikely to completely eradicate cccDNA, permanent silencing of cccDNA transcription should be prioritized (Revill et al., 2019). The regulatory function of HBx is of great importance in the transcription of cccDNA, and the vital role of HBx in HBV pathogenesis might lead to the discovery of antiviral drugs against HBx.

In our previous small-molecule compound screening, we found that rapamycin could inhibit the exogenous expression of HBx protein in Huh-7 cells, which caught our attention (Cheng et al., 2021). In 1964, rapamycin was first found in soil samples collected from Rapa Nui (Easter Island). From then on, it was confirmed to have outstanding immunosuppressive, antitumor, and antifungal properties (clinically referred to as sirolimus) (Vezina et al., 1975; Martel et al., 1977; Eng et al., 1984). After further research, some of its effects were found to be mediated by inhibiting the signal transduction for cell growth as well as proliferation (Chung et al., 1992). Moreover, the complete mechanism of rapamycin was illuminated after the mechanistic target of rapamycin (mTOR) was identified (Brown et al., 1994; Sabers et al., 1995). The mTOR protein is one of the serine/threonine protein kinases from the PI3K-related kinase (PIKK) family and it forms three individual protein complexes named mTOR complex 1, 2, and 3 (mTORC1/2/3) (Yin et al., 2016; Harwood et al., 2018). mTOR complexes are the core components of major eukaryotic signaling networks and they coordinate cell growth in complicated environmental conditions, playing a fundamental role in cell and organismal physiology, such as glucose homeostasis, adipogenesis, immune function, energy sensing, and DNA damage (Saxton and Sabatini, 2017). In addition, intensive activation of mTORC1 is occurred in many oncogenes such as PI3K and AKT and in tumor suppressors such as LKB1, TSC1/2, and PTEN (Li et al., 2014). Temsirolimus and everolimus are the derivatives of the initial rapamycin and were both approved for the treatment of multiple diseases such as advanced renal cancer carcinoma (RCC), neuroendocrine carcinoma (NEC), and endometrial cancer (Li et al., 2014). Moreover, numerous studies have shown that rapamycin inhibits the replication of many viruses such as porcine epidemic diarrhea virus, Zika virus, human immunodeficiency virus type 1, influenza A virus, Epstein–Barr virus, and even COVID-19 (Roy et al., 2002; Krams and Martinez, 2008; Ko et al., 2017; Wang et al., 2019; Husain and Byrareddy, 2020; Sahoo et al., 2020). However, whether rapamycin has an effect on HBV infection and CHB remains to be further studied.

In this study, we found that rapamycin reduced HBx protein stability by promoting its ubiquitination. More importantly, rapamycin has an antiviral effect against HBV RNAs and DNA, as well as activity against cccDNA transcription. Similar results were also observed in a recombinant cccDNA mouse model. In summary, we authorized the effect of rapamycin on HBV cccDNA transcription and HBV replication, providing the research evidence for the development of effective new HBV treatment drugs.

## Materials and methods

### Cells

HepG2 cells were purchased from the American Type Culture Collection, and the stable cell line HepG2-NTCP (Na^+^/taurocholate cotransporting polypeptide) was constructed by our laboratory. Huh-7 cells were acquired from the Health Science Research Resource Bank. Primary human hepatocytes (PHHs) were obtained from ScienCell Research Laboratories. HepAD38 cells were presented by professor Ning-shao Xia (Xiamen University, China) and were maintained in Dulbecco’s modified Eagle’s medium (DMEM, Sigma-Aldrich, St.Louis, MO, United States) added with 10% fetal bovine serum (FBS, LONSERA, Shanghai, China) and 400 μg/ml G418 (BIOFROXX, Einhausen, Germany). HepG2-NTCP and Huh-7 cells were cultured in DMEM with 10% fetal bovine serum and 1% penicillin-streptomycin (HyClone, Logan, UT, United States). PHHs were cultured in a hepatocyte medium (ScienCell, San Diego, CA, United States). All the cells were incubated in the incubator at 37°C with 5% CO_2_.

### Plasmids and drugs

pCH9/3091 plasmid (HBV1.1 plasmid, which contains 1.1 copies of the HBV genome driven by the CMV promoter) was presented by professor Lin Lan (Army Medical University, China). pGEM-HBV1.3 plasmid (harbors 1.3 copies of the HBV genome driven by the HBV promoter) was presented by professor U. Protzer (University of Heidelberg, Germany). pcDNA3.1-2 × HA-HBx was kindly provided by Prof. Jieliang Chen (Fudan University, Shanghai, China). HBc, HBx, HBs, and viral polymerase (Pol) were recombined by inserting the indicated coding sequence into pcDNA3.1 (harbors a 3 × Flag tag). The 3 × Flag-HBx-K95R plasmid was constructed by mutating the 95th amino acid from lysine to arginine based on 3 × Flag-HBx by PCR, and the primer sequences are shown in Supplementary Table. The HBx deletion HBV plasmid (HBV1.1-Δx) was structured by recommending a stop codon in the front of the HBx sequence based on HBV1.1. The siRNA of mTOR was purchased from Tsingke, China (Lot Number: D107130961-D107130964). Rapamycin (CAS Number: 53123-88-9) was obtained from MedChemExpress (Monmouth Junction, NJ, United States) and entecavir (CAS Number: 142217-69-4) was purchased from Selleck (Houston, United States). Cycloheximide was purchased from Amresco (97064-722, WA, United States), and proteasome inhibitor MG132 (S2619) and Bortezomib (S1013) were acquired from Selleck Chemicals (Houston, United States).

### Plasmid transfection

Cells were transfected with indicated plasmids by using the Lipofectamine^®^ 3,000 transfection kit (Invitrogen, Walsham, MA, United States) on the ground of the manufacturer’s instructions.

### Antibodies

The rabbit anti-20S (PSMB9) polyclonal antibody (14544-1-AP) and rabbit anti-26S (PSMD2) polyclonal antibody (14748-1-AP) were purchased from Proteintech (Chicago, United States). The rabbit anti-mTOR (7C10) monoclonal antibody (#2983T), anti-Flag antibody (#14793S), and mouse anti-Ubiquitin antibody (#3936) were acquired from Cell Signaling Technology (CST, Boston, United States). The rabbit anti-HBcAg (B0586) was purchased from Dako (Copenhagen, Denmark). The Rabbit IgG (NI01) was acquired from Millipore (Darmstadt, Germany) and the mouse anti-HBxAg (1844) was purchased from ViroStat (ME, United States). The mouse anti-GAPDH (1A6) monoclonal antibody (MB001) was obtained from Bioworld (Bloomington, United States). The rabbit anti-HBsAg (NB100-62652) was purchased from Novus (CO, United States). The IPKine™ HRP anti-mouse IgG LCS antibody was obtained from Abbkine (Wuhan, China).

### Viruses and infection

Normally, HBV viruses were enriched from the HepAD38 cells culture medium by precipitating with 5% polyethylene glycol 8000 (PEG8000). Wild-type HBV (HBV WT) particles were assembled from the supernatant of the culture medium of Huh-7 cells transfected with pHBV1.1, and correspondingly, the HBx-deficient HBV particles (HBV-Δx) were concentrated from the epipelagic medium of Huh-7 cells co-transfected with pHBV1.1-ΔHBx and an HBx-expressing vector. The HBV viral particles were quantified as the depictive method previously (Yan et al., 2015). In brief, the HBV DNA in HBV particles was extracted and quantified by absolute real-time PCR as described below. Different concentrations of pHBV1.1 (3 × 10^3^, 3 × 10^4^, 3 × 10^5^, 3 × 10^6^, and 3 × 10^7^ copies/μl) were served as the template to generate a standard curve used for absolute real-time PCR quantification. For HBV infection, HepG2-NTCP cells or PHHs were infected with 1,000 vge HBV and incubated for 1 day with 4% PEG8000. Next, the cells were placed in a culture medium supplemented with 2.5% dimethyl sulfoxide (DMSO) for further experiments after being washed with phosphate buffer saline (PBS). Finally, cells were treated with the appropriate concentration of rapamycin 2 days after infection with the virus.

### MTT assay

The thiazolyl blue tetrazolium bromide assay (MTT assay) was performed to determine the cytotoxicity of rapamycin on diversified cells. In brief, cells were exposed to rapamycin in a series of concentrations. After incubation for 3 days (Huh-7 cells) or 6 days (PHHs and HepG2-NTCP cells), MTT (5 mg/ml) was transferred into the medium and incubated avoiding light for 4 h. Finally, the medium was sucked away and replaced with 100 μl DMSO. The OD_490_ of all wells was detected, and the CC50 was calculated by non-linear regression.

### Alamar blue assay

The Alamar blue assay was carried out according to the instruction book with the Blue™ Cell Viability Reagent (Invitrogen, United States). In brief, the medium on days 2, 4, and 6 was removed from the culture plate after rapamycin treatment and replaced by a 1× mix working solution. After 2 h of incubation protected from light, the excitation wavelength and emission wavelength were detected at 560 and 590 nm, respectively.

### Nano-Glo^®^ HiBiT Lytic assay

To screen small-molecule compounds targeting HBx, we used the Nano-Glo^®^ HiBiT Lytic system (N3030, Promega, WI, United States) according to the specification and as described in detail previously (Cheng et al., 2021). In brief, the lytic reagent was used to dilute the lytic substrate (1:50) and the LgBiT protein (1:100) into the lytic buffer. Then, the lytic reagent was transferred into the wells containing cells transfected with the HiBiT-HBx plasmid. Finally, the expression level of HBx was evaluated by fluorescence intensity.

### Co-immunoprecipitation and western blotting analysis

Cells were lysed in IP lysis buffer supplemented with an appropriate protease inhibitor. Then, the BCA protein assay kit was used for determining the protein concentration after centrifugation (Thermo Scientific, MA, United States), and 500 μg protein was incubated with protein A/G magnetic beads (Millipore, Germany). Next, the beads and protein were immunoprecipitated with the indicated antibodies at 4°C overnight. As for western blotting, the samples by SDS-PAGE were performed for separating the proteins and the proteins were transferred to a PVDF membrane (GE Healthcare, Buckinghamshire, United Kingdom). About 5% non-fat milk was prepared for blocking and the membrane was incubated with indicated antibody. Next day, HRP-conjugated secondary antibody was added to 5% non-fat milk and incubated with proteins. The signals were collected by western blotting analysis reagents (Millipore, MA, United States).

### Enzyme-linked immunosorbent assay

The HBsAg and HBeAg levels in the cell supernatant medium were detected *via* enzyme-linked immunosorbent assay kits (KHB, Shanghai, China) referring to the specification. Samples were added to 96-well plates, and the absorbances at 450 nm wavelength were detected.

### Hepatitis B virus core DNA extraction and quantitation

Cells were lysed in HBV core DNA lysis buffer (10 mM pH 8.0 Tris-HCl, 1% NP-40, 1 mM ethylene diamine tetraacetic acid (EDTA), 2% sucrose), and the nuclei were removed by centrifugation. Then, the nucleic acids were digested by 10 mM MgCl_2_ and 40 IU/mL DNase I followed by 5% PEG8000 for precipitation and added 500 μg/ml proteinase K. Finally, the samples were purified and extracted with phenol/chloroform. For quantitative PCR analysis, the Fast Start Universal SYBR Green Master Mix (06924204001, Roche, Mannheim, Germany) was used for quantification.

### Hirt covalently closed circular DNA extraction and Taq-man probe qRT–PCR

For HBV cccDNA extraction, we used the previously described modified Hirt method (Cheng et al., 2021). In brief, the cells were lysed in SDS lysis buffer and then added 125 μl of 2.5-M KCl overnight. After centrifugation, the supernatant was purified by phenol/chloroform. Then, HBV cccDNA was monitored by qRT–PCR using the TaqMan probe in the following steps. First, the samples were heated at 80°C for 5 min and transferred on the ice immediately. Second, the samples were digested with exonuclease V (M0345S, New England Biolabs, MA, United States). Third, the cccDNA samples were heated at 100°C for 20 min. Then, Taq-man probe qRT–PCR was performed for cccDNA quantification.

### Southern and northern blotting analysis

Southern blotting and northern blotting analysis were performed by employing the DIG-High Prime DNA Labeling Detection Starter Kit (11585614910, Roche, Germany) and the DIG Northern Starter Kit (12039672910, Roche, Germany). First, the DNA or RNA samples were separated by running a 1% agarose gel, and then the DNA or RNA were transferred to the Nylon membrane (Roche, Germany). Then, the UV crosslinking was performed, and the membrane was hybridized with the digoxigenin-labeled DNA probe (a sequence of the HBV genome, D type) or an RNA probe (1,000 bp, plus a strand-specific HBV probe, HBV genome 126–1,225 bp) at 42 or 68°C overnight. After incubating with the indicted anti-digoxin antibody, the signals were collected by using the X-ray films.

### Hepatitis B virus RNA extraction and reverse-transcription PCR

The total RNA of cells was extracted by TRIzol Universal Reagent (DP424, TIANGEN, Beijing, China) according to the user manual. About 1 μg RNA was reverse transcripted with the FastKing RT Kit (KR116-02, TIANGEN, Beijing, China). The relative RNA expression level was quantified by RT–PCR. Besides, the mRNA level of β-actin was measured as the loading control, and the data were processed by the 2^–ΔΔ*Ct*^ method.

### Animal experiment

C57BL/6 male mice aged approximately 6–8 weeks were selected and injected hydrodynamically with 4 μg precursor recombinant cccDNA (prcccDNA) and 4 μg pCMV-KRAB-Cre plasmid (contains a KRAB-Cre fusion recombinase) through the tail vein. These two plasmids could form recombinant cccDNA (rcccDNA) with a similar structure to the natural cccDNA and were kindly presented by Prof.Qiang Deng (Ruijin Hospital, Shanghai Jiao Tong University School of Medicine), and for the detailed information and principles about theses plasmids, refer to Qi et al. (2014) and Li et al. (2018). Besides, it was reported that HBsAg and HBcAg could be observed by immunofluorescence in liver tissue of rcccDNA mice 3 days after hydrodynamic injection of these plasmids and they exhibited HBV antigenicity for up to 9 weeks (Qi et al., 2014). Moreover, the KRAB-Cre fusion can induce a higher level of rcccDNA (Li et al., 2018). After 1 week, the blood was drawn from the tail veins, then the HBV DNA level in serum was quantified by qPCR to confirm whether this mouse model was constructed effectively. Then, the successful mice were divided into four groups of five mice/group randomly and treated with 0.9% saline (negative control), 0.02 mg/kg ETV (positive control), 1 mg/kg rapamycin, or both drugs by gavage every 2 days for 20 days, respectively. The dose of the drug used was as described in previous studies (Huynh et al., 2008; Huynh, 2010; Ren et al., 2019).

### Alanine aminotransferase and aspartate aminotransferase assays

The level of alanine aminotransferase (ALT) and aspartate aminotransferase (AST) were detected by indicted kits (C009-2-1/C010-2-1, Jiancheng, Nanjing, China) referring to the instructions.

### Statistical analysis

The results are expressed as the mean ± SD. All statistical analyses were carried out by the non-parametric Mann–Whitney *U* test or by one-way ANOVA, and *P* < 0.05 was considered statistically significant (*P < 0.05).

## Results

### Rapamycin reduced HBx expression

**FIGURE 1:**
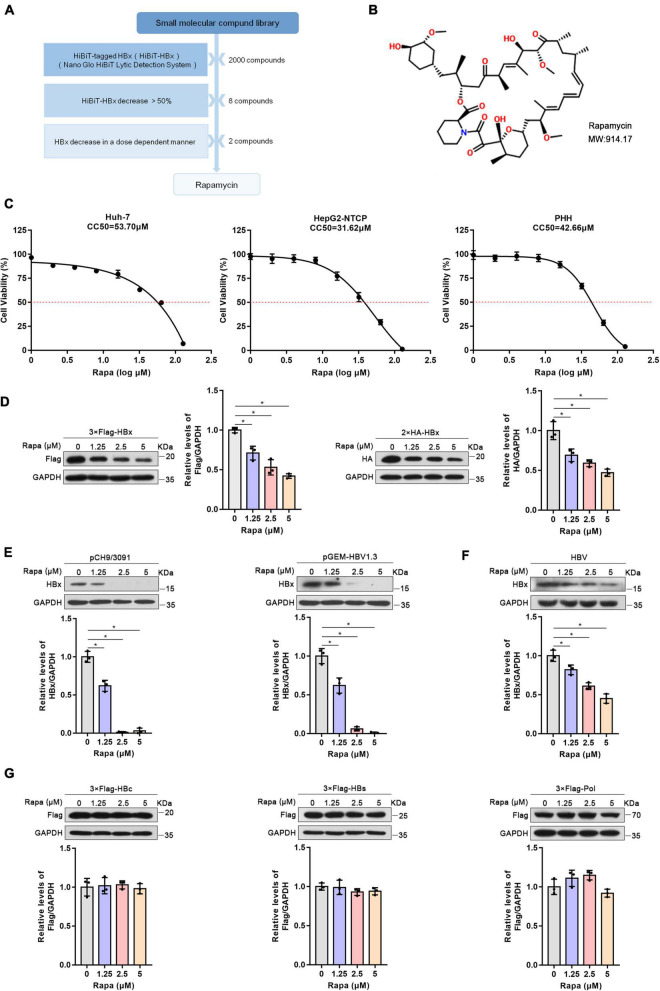
Rapamycin reduced HBx expression. **(A)** Schematic process of screening small-molecule compounds targeting HBx. **(B)** The chemical structure of rapamycin. **(C)** Effects of rapamycin on the viability of different cells were determined by the MTT assay after treatment with the indicated concentrations of rapamycin for 3 days (Huh-7 cells) or 6 days (HepG2-NTCP and PHH cells). **(D,E)** Huh-7 cells were transfected with 3 × Flag-HBx, 2 × HA-HBx, pCH9/3091, or pGEM-HBV1.3 plasmid for 24 h and were then treated with rapamycin for 24 h. **(F)** HepG2-NTCP cells were infected with 10^3^ genome equivalents/cells of HBV particles and then treated with rapamycin for 6 days. **(G)** Huh-7 cells were transfected with 3 × Flag-HBc, 3 × Flag-HBs, or 3 × Flag-Pol plasmids and were then treated with rapamycin for 1 day. The level of the corresponding protein was examined by western blotting analysis, and GAPDH was used as the loading control. Cell lysates were subjected to immunoprecipitation with anti-Flag antibody and then immunoblotted with anti-ubiquitin antibody. The data are presented as the mean ± SD of three independent experiments.

HiBiT-HBx was previously constructed to screen small-molecule compounds targeting HBx by the HiBiT lytic detection system (Cheng et al., 2021). After screening, rapamycin, an inhibitor of mTOR, was considered a candidate for further research (Figure 1A). The chemical structure of rapamycin is shown in Figure 1B. The MTT assay showed that the CC50 values of rapamycin in Huh-7, HepG2-NTCP, and PHH cells were 53.7, 31.62, and 42.66 μM, respectively (Figure 1C). We chose 0, 1.25, 2.5, and 5 μM for the follow-up experiments, and the Alamar blue experiment showed that the above concentrations of drugs did not affect the cell activity (Supplementary Figure 1).

To verify the repressive effect of rapamycin on HBx protein, we constructed plasmids with 3 × Flag and 2 × HA-tagged HBx plasmids. It was found that rapamycin treatment resulted in a decrease of HBx protein dose-dependently in Huh-7 cells (Figure 1D). In addition, rapamycin treatment also decreased the protein level of HBx in a dose-dependent manner in pHBV1.1 or pGEM-HBV1.3-transfected Huh-7 cells (Figure 1E). Moreover, rapamycin also decreased the HBx protein level by 55% under 5 μM rapamycin treatment in HepG2-NTCP cells infected with HBV (Figure 1F). Interestingly, other HBV proteins such as P polymerase, HBc, and HBs proteins, were not affected by rapamycin treatment (Figure 1G).

### Rapamycin inhibited the HBx protein level by decreasing its stability

**FIGURE 2:**
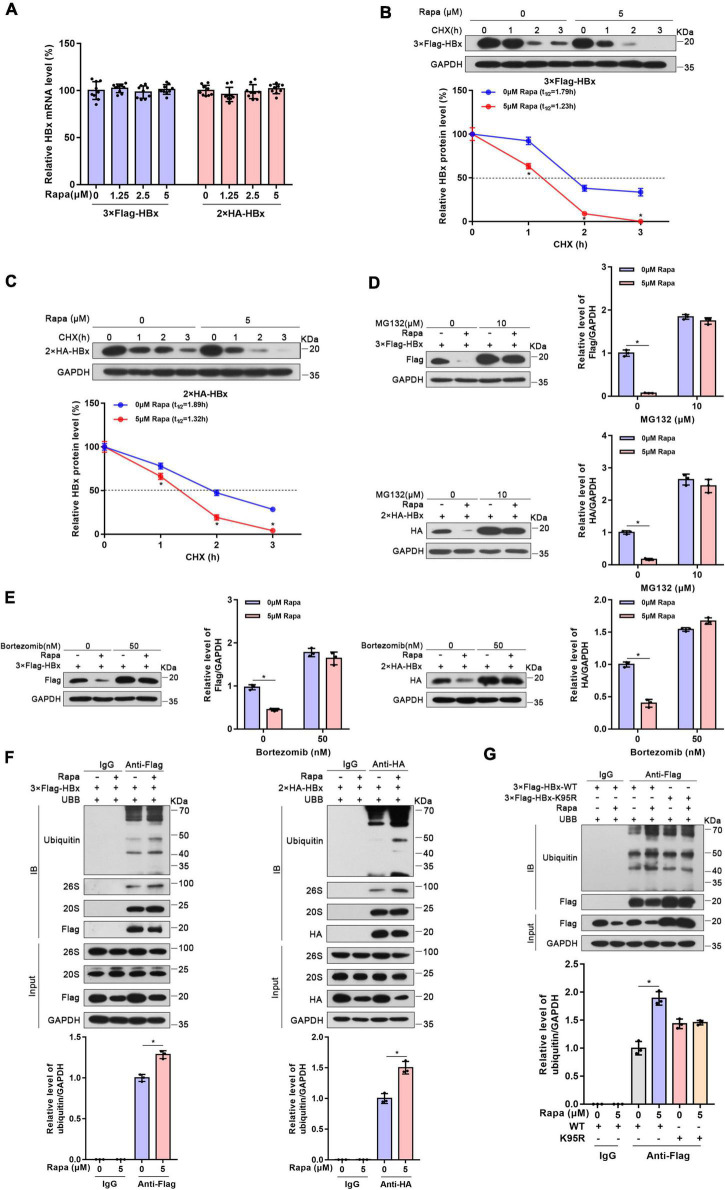
Rapamycin promoted the degradation of HBx *via* the ubiquitin-proteasome system. Huh-7 cells were transfected with the corresponding plasmid and then treated with rapamycin for 24 h. **(A)** The mRNA levels of HBx were examined by real-time PCR using specific primers in Huh7 cells transfected with 3 × Flag-HBx or 2 × HA plasmid. The β-actin mRNA level was used as an internal control. **(B,C)** Huh-7 cells transfected with 3 × Flag-HBx or 2 × HA-HBx plasmid for 24 h were then exposed to 5 μM rapamycin and 10 μg/ml cycloheximide for the indicated times. The levels of the proteins were examined by western blotting analysis, and GAPDH was used as the loading control. **(D,E)** Ubiquitin-proteasome inhibitor MG132 or bortezomib eliminated the inhibiting effect of rapamycin on HBx. Huh-7 cells transfected with 3 × Flag-HBx or 2 × HA-HBx plasmid were exposed to 5 μM rapamycin for 24 h, and MG132 or bortezomib was added 8 h before the cells were harvested. The levels of the proteins were examined by western blotting analysis, and GAPDH was used as the loading control. **(F)** Huh-7 cells were co-transfected with 3 × Flag-HBx or 2 × HA-HBx and Ubb plasmids and then treated with 5 μM rapamycin for 24 h. Cell lysates were subjected to coimmunoprecipitation with anti-Flag or anti-HA antibody and were then immunoblotted with anti-ubiquitin antibody. **(G)** Huh-7 cells were co-transfected with 3 × Flag-HBx-WT or 3 × Flag-HBx-K95R and Ubb plasmids and were then treated with 5 μM rapamycin for 24 h. Protein levels were analyzed after Co-IP. The data are presented as the mean ± SD of three independent experiments. *P < 0.05.

To elucidate how rapamycin decreases HBx protein levels, RNA extraction and RT–PCR were performed. We found that rapamycin treatment did not change the level of HBx mRNA in Huh-7 cells transfected with 2 × HA-HBx or 3 × Flag-HBx plasmid (Figure 2A). The impression of rapamycin on the stability of HBx protein was confirmed by cycloheximide assay. We found that the stability of HBx was reduced notably after exposure to rapamycin (Figures 2B,C). In addition, the stability of HBs, HBc, and P polymerase was not affected by rapamycin treatment (Supplementary Figure 2). In conclusion, rapamycin treatment reduced the stability of the HBx protein, which caused a decrease in the HBx protein level.

### Rapamycin enhanced the ubiquitination of HBx

It was well-known that the ubiquitin-proteasome system (UPS) is one of the main methods of eukaryotic cells to degrade proteins. Importantly, mTOR inhibition could raise the levels of K48-linked ubiquitination and promote to degrade proteins in cells (Zhao et al., 2015). Moreover, HBx protein was reported to interact with many UPS proteins such as DDB1, CSN, and proteasome (Minor and Slagle, 2014). Therefore, we chose two proteasome inhibitors, MG132 and bortezomib, which inhibit 26S and 20S proteasomal activities. As expected, the inhibition of HBx protein by rapamycin was almost eliminated after proteasome inhibitors treatment (Figures 2D,E). Moreover, the inhibition of endogenous HBx protein by rapamycin was also eliminated after proteasome inhibitors treatment in HBV-infected HepG2-NTCP cells (Supplementary Figure 3). These results proved that the UPS may show an indispensable role in the degradation of HBx caused by rapamycin.

Then, we detected the ubiquitination level of HBx protein through coimmunoprecipitation experiments. These results indicated that the ubiquitination level of HBx increased significantly after rapamycin treatment. Moreover, we found that HBx could bind to the 20S and 26S proteasomes (Figure 2F). It was reported that ubiquitination site K95 shows a key function in HBx ubiquitination (Tan et al., 2019). Therefore, to determine whether the reduction in HBx protein levels by rapamycin depends on ubiquitination, we mutated this ubiquitination site and found that rapamycin did not change the ubiquitination levels of mutant HBx (Figure 2G). These results suggested that the role of rapamycin in reducing HBx protein levels is dependent on the UPS.

### Rapamycin blocked hepatitis B virus covalently closed circular DNA transcription

**FIGURE 3:**
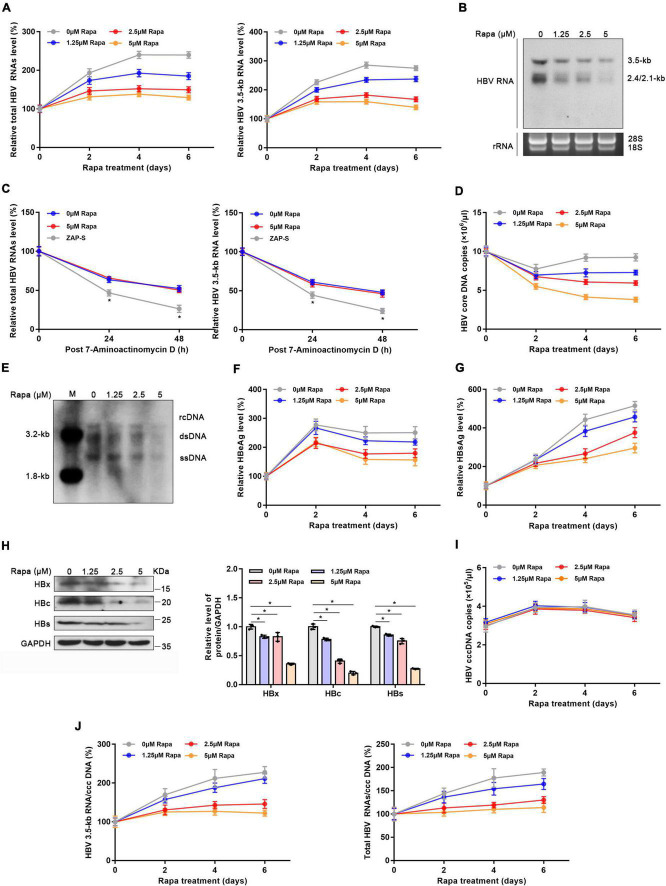
Rapamycin blocked cccDNA transcription in HBV-infected HepG2-NTCP cells. HepG2-NTCP cells were infected with 10^3^ genome equivalents/cells of HBV particles in the presence of 4% PEG8000 for 24 h. Two days after infection, the cells were treated with the appropriate concentration of rapamycin for 6 days. **(A)** The levels of total HBV RNAs and 3.5-kb RNA were detected by real-time PCR using specific primers. The β-actin mRNA level was used as an internal control. **(B)** Northern blotting hybridization of HBV RNAs by using a DIG-labeled HBV riboprobe. Ribosomal RNAs (28 and 18 s) served as the loading control. **(C)** HBV-infected cells were treated with 7-aminoactinomycin D (5 μg/ml) for 24 or 48 h. HBV RNAs were quantified by real-time PCR. ZAP-S was used as a positive control. **(D,E)** The level of HBV core DNA was analyzed by real-time PCR analysis and Southern blotting analysis. **(F,G)** The levels of secreted HBsAg and HBeAg in the cell culture supernatant were detected by ELISA. **(H)** The levels of HBx, HBc, and HBs proteins were detected by western blotting analysis, and GAPDH was used as the loading control. **(I,J)** Total HBV RNA, 3.5-kb RNA, and cccDNA levels were analyzed by real-time PCR and were then used to calculate the ratio of total RNA/cccDNA or 3.5-kb RNA/cccDNA. The data are presented as the mean ± SD of three independent experiments. *P < 0.05.

Given the meaningful function of rapamycin in regulating HBx, we explored whether rapamycin has an inhibiting effect on cccDNA transcription. After 5 μM rapamycin treatment for 6 days, total HBV RNA level and 3.5-kb RNA were decreased by 46 and 49%, respectively (Figure 3A). Decreased HBV RNAs levels were confirmed by northern blotting analysis (Figure 3B). In addition, rapamycin did not affect the stability of HBV RNA (Figure 3C), and zinc finger antiviral protein S isoform (ZAP-S)-mediated RNA decay served as a positive control (Guo et al., 2007; Mao et al., 2013; Zhou et al., 2019). The HBV core DNA level was also reduced by 58% under 5 μM rapamycin treatment for 6 days, as well as the qPCR (Figure 3D), and Southern blotting analysis proved this result (Figure 3E). HBeAg and HBsAg suppression was also observed after rapamycin treatment (Figures 3F,G). In addition, western blotting analysis showed that rapamycin had a significant inhibitory effect on HBc, HBs, and HBx proteins (Figure 3H). Although the level of cccDNA was not changed, the ratios of total HBV RNAs/cccDNA and 3.5-kb RNA/cccDNA, which indicate transcriptional activity of cccDNA, were notably decreased by 46 and 39.8%, respectively (Figures 3I,J). Moreover, similar results were also obtained in PHH cells (Supplementary Figure 4).

It was previously reported that rapamycin could inhibit HBV infection by blocking viral attachment to the host cell surface, which was proven by AlphaScreen technology targeting the envelope-receptor interaction (Saso et al., 2018). To study whether rapamycin also inhibits processes other than the entry process, Huh-7 cells were co-transfected with prcccDNA and pCMV-KRAB-Cre plasmids which could directly form (r)cccDNA in cells (Qi et al., 2014; Li et al., 2018), followed by treatment with rapamycin. Interestingly, rapamycin significantly decreased the levels of total HBV RNAs, HBV 3.5-kb RNA, HBV core DNA, HBsAg, and HBeAg (Supplementary Figures 5A–C). In addition, western blotting analysis also proved that rapamycin showed a notable inhibitory effect on HBc, HBs, and HBx proteins (Supplementary Figure 5D). In summary, rapamycin could reduce the transcription level of cccDNA.

### The blockade of rapamycin on hepatitis B virus covalently closed circular DNA transcription is dependent on HBx ubiquitination

**FIGURE 4:**
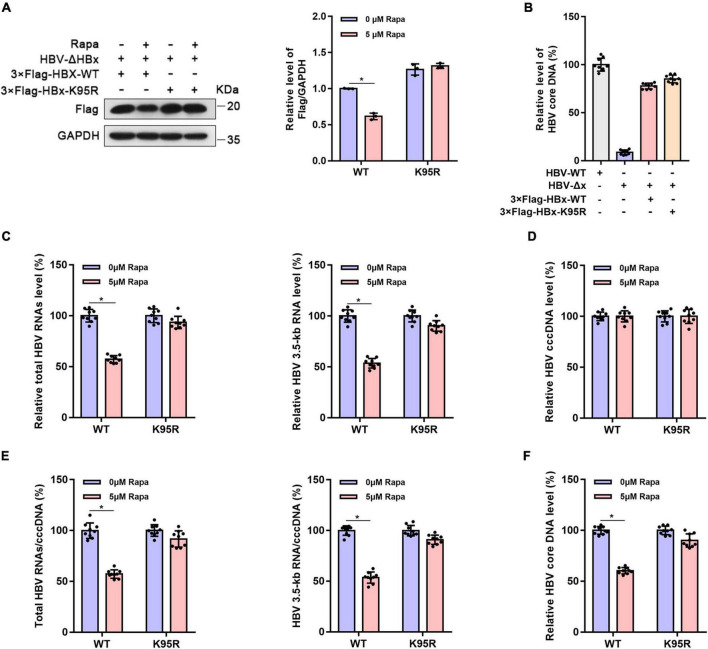
The inhibitor effect of rapamycin on HBV cccDNA transcription is dependent on ubiquitination. HepG2-NTCP cells were infected with 10^3^ genome equivalents/cells of HBV-ΔHBx particles and were then transfected with 3 × Flag-HBx-WT or 3 × Flag-HBx-K95R plasmid. Cells were exposed to rapamycin for 6 days. **(A)** The protein level of HBx was detected by western blotting analysis, and GAPDH was used as the loading control. **(B)** The levels of virus production after HBx complementation are represented by the HBV core DNA. The level of HBV core DNA was analyzed by real-time PCR. **(C)** The levels of total HBV RNAs and 3.5-kb RNA were detected by real-time PCR using specific primers. The β-actin mRNA level was used as an internal control. **(D,E)** The cccDNA level was analyzed by real-time PCR, and the HBV 3.5-kb RNA/cccDNA and total RNA/cccDNA ratios were calculated. **(F)** The level of HBV core DNA was analyzed by real-time PCR. The data are presented as the mean ± SD of three independent experiments. *P < 0.05.

To verify whether the inhibition of rapamycin on HBV cccDNA transcription is dependent on HBx ubiquitination, HepG2-NTCP cells were infected with HBx-deleted HBV particles (HBV-Δx) and were transfected with wild-type 3 × Flag-HBx-WT or 95th ubiquitination site-mutated 3 × Flag-HBx-K95R plasmid and then treated with rapamycin (Figure 4A). The levels of virus production after HBx complementation were represented by HBV core DNA level, and the levels were approximately the same after complementarity of wild-type and mutant HBx (Figure 4B). The levels of total HBV RNAs (Figure 4C), HBV 3.5-kb RNA (Figure 4C), cccDNA (Figure 4D), the ratio of total HBV RNAs/cccDNA and HBV 3.5-kb RNA/cccDNA (Figure 4E), and the level of HBV core DNA (Figure 4F) were detected. Interestingly, the inhibition of rapamycin on cccDNA transcription was abolished when the 95th ubiquitination site of HBx was mutated compared with that in wild-type HBx-transfected cells. In summary, HBx ubiquitination showed an indispensable role in the inhibitory effect of rapamycin on cccDNA transcription.

### Mechanistic target of rapamycin knockdown reduced hepatitis B virus transcription and replication

**FIGURE 5:**
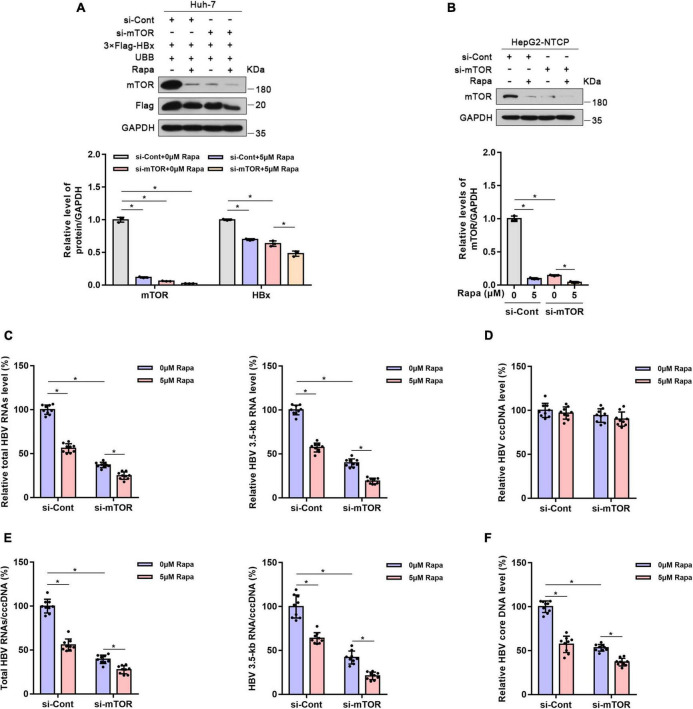
Mechanistic target of rapamycin (mTOR) knockdown reduced HBV transcription and replication. Huh-7 cells were transfected with 3 × Flag-HBx and UBB plasmids and were then transfected with siRNAs for mTOR. Finally, the cells were treated with 5 μM rapamycin for 24 h. HepG2-NTCP cells were transfected with siRNAs for mTOR, infected with 10^3^ genome equivalents/cells of HBV, and then exposed to rapamycin for 6 days. **(A,B)** The levels of the different proteins in Huh-7 cells or HepG2-NTCP cells were examined by western blotting analysis, and GAPDH served as the loading control. **(C)** The levels of total HBV RNAs and 3.5-kb RNA were detected by real-time PCR using specific primers. The β-actin mRNA level was used as an internal control. **(D,E)** The level of cccDNA was analyzed by real-time PCR, and the ratios of HBV 3.5-kb RNA/cccDNA and total RNAs/cccDNA were calculated. **(F)** The level of HBV core DNA was analyzed by real-time PCR. The data are presented as the mean ± SD of three independent experiments. *P < 0.05.

The mTOR protein is the direct target of rapamycin, and mTOR plays an important role in controlling protein levels (Brown et al., 1994; Sabers et al., 1995; Saxton and Sabatini, 2017). Thus, we wondered whether the inhibition of HBV cccDNA transcription by rapamycin is related to mTOR. As expected, mTOR knockdown reduced HBx protein expression in Huh-7 cells co-transfected with 3 × Flag-HBx and UBB plasmids (Figure 5A). Meanwhile, mTOR knockdown significantly increased the ubiquitination level of HBx protein (Supplementary Figure 6). Furthermore, the levels of total HBV RNAs, HBV 3.5-kb RNA, HBV core DNA, and the ratio of total HBV RNAs/cccDNA and HBV 3.5-kb RNA/cccDNA were decreased significantly (Figures 5B–F). This proved that mTOR knockdown reduced HBV transcription and replication.

### Rapamycin showed effective antiviral activity against hepatitis B virus in (r)cccDNA mice

**FIGURE 6:**
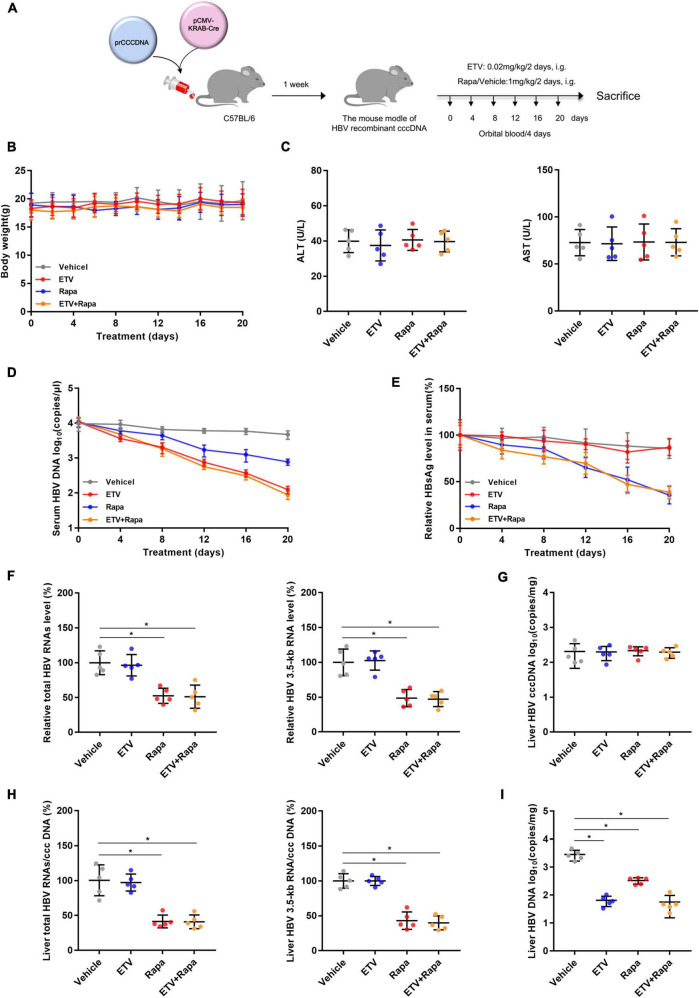
Rapamycin showed effective antiviral activity against HBV in (r)cccDNA mice. **(A)** A flow chart explaining the method and concentration of ETV and rapamycin as well as the interval of orbital blood collection. The mouse model was established by injecting 4 μg prcccDNA and 4 μg pCMV-KRAB-Cre into the tail vein, and models with HBV infection involving HBV (r)cccDNA that were constructed successfully and were randomly assigned to four groups (*n* = 5 per group). Blood samples were collected every 4 days for 20 days. **(B)** The mouse’s body weight was measured by an electronic scale. **(C)** ALT and AST were detected by corresponding kits using a microplate reader. **(D)** The level of HBV DNA in the serum was analyzed by real-time PCR. **(E)** HBsAg in the serum was measured by an ELISA kit. **(F–H)** The levels of total HBV RNAs, 3.5-kb RNA, and cccDNA were analyzed by real-time PCR, and the ratios of HBV 3.5-kb RNA/cccDNA and total RNAs/cccDNA were calculated. **(I)** The level of HBV DNA in the liver was analyzed by real-time PCR. The data are presented as the mean ± SD. *P < 0.05.

To evaluate whether rapamycin could suppress HBV replication *in vivo*, we chose the recombinant cccDNA (rcccDNA) mouse model as described previously (Qi et al., 2014). The rcccDNA mice were treated with vehicle or rapamycin at 1 mg/kg and/or ETV 0.02 mg/kg once every 2 days by gavage for 20 days (Figure 6A). There was no obvious difference in the body weight between treatment groups as monitored every 2 days (Figure 6B), and the levels of serum ALT and AST in different groups showed no obvious differences (Figure 6C). Rapamycin reduced both serum HBV DNA and HBsAg levels (Figures 6D,E). In addition, mice of all groups were sacrificed to collect the livers after treating by rapamycin for 20 days. Decreased levels of intrahepatic total HBV RNAs, HBV 3.5-kb RNA, HBV DNA, and the ratio of total HBV RNAs/cccDNA and HBV 3.5-kb RNA/cccDNA were observed in the rapamycin-treated group compared to the vehicle group (Figures 6F–I). Importantly, the ratios of total HBV RNAs/cccDNA and HBV 3.5-kb RNA/cccDNA were decreased by 59 and 57%, respectively, in the rapamycin treatment group on Day 20. These data showed that rapamycin has effective anti-HBV activity *in vivo*.

## Discussion

A complete cure of HBV is still the ultimate objective of anti-HBV therapy, and it is necessary to combine new antiviral methods to reduce the large burden of CHB (Gane, 2017). The elimination of HBV cccDNA is the unquestionable and most efficient strategy to cure HBV infection (Revill et al., 2019). However, interferon and nucleos(t)ide analogs used for current antiviral therapy against chronic HBV infection are unable to eliminate cccDNA. Therefore, new drugs and antiviral strategies targeting cccDNA are of great significance.

Since it is not feasible to complete the elimination cccDNA now (Yang and Kao, 2014), cccDNA transcriptional silence can play a vital role in controlling HBV. Importantly, control of cccDNA transcription might be achieved by targeting HBV viral protein HBx, which plays a critical role in HBV replication as well as cccDNA epigenetic modification and stability (Belloni et al., 2009; Levrero et al., 2009; Lucifora et al., 2011). Moreover, it was reported that HBx relieved the repression of cccDNA transcription caused by PRMT5 and SETDB1 (Riviere et al., 2015; Zhang et al., 2017). Besides, HBx can also bind to lncRNA DLEU2 to maintain the transcription of cccDNA and other host cancer-related genes (Salerno et al., 2020). Moreover, it has been reported that HBx can promote the degradation of the smc5/6 complex to relieve its inhibition of cccDNA transcription (Decorsiere et al., 2016; Murphy et al., 2016; Livingston et al., 2017). Therefore, HBx may serve as a possible antiviral target to inhibit the transcription of cccDNA and HBV replication.

Several studies have shown that increasing HBx stability enhances its activation of cccDNA transcription (Su et al., 2017; Chen et al., 2019; Tan et al., 2019). Our group used the established HiBiT-HBx detection system and identified that DIC could inhibit HBx expression by ubiquitination-independent 20S proteasome-mediated cleavage (Cheng et al., 2021). Similarly, Chen et al. (2019) reported that the chromatin remodeling factor BAF155 can protect HBx from UPS degradation. Su et al. (2017) also found that deubiquitylation of HBx enhances the stability and transactivation activity of HBx. In this study, we identified rapamycin as another small-molecule compound that could inhibit the level of HBx protein in Huh-7 cells transfected with 3 × Flag-HBx, 2 × HA-HBx, pCH9/3091, and pGEM-HBV1.3, even in HBV-infected HepG2-NTCP cells. Further studies conclusively certified that rapamycin targets HBx to decrease its stability by promoting ubiquitin-dependent proteasomal degradation.

In view of the indispensable role of HBx in cccDNA transcription, we examined various indicators during the HBV life cycle after rapamycin treatment and found that rapamycin could suppress the levels of total HBV RNAs, HBV 3.5-kb RNA, and HBV core DNA, as well as the ratios of total HBV RNAs/cccDNA and HBV 3.5-kb RNA/cccDNA. In addition, further research revealed that this effect of rapamycin depends on HBx. Moreover, similar effects of rapamycin on HBV cccDNA transcription were also confirmed in the recombinant cccDNA mouse model. Together, these results suggested that rapamycin has evident antiviral activity both *in vitro* and *in vivo*.

However, one investigation showed inconsistent results. Guo et al. (2007) found that rapamycin treatment increased the HBV RNA and HBV DNA levels in HepG2.2.15 cells. In HepG2.2.15 cells, the main source of HBV RNA and DNA is the HBV genome integrated into the host genome, while in our HBV-infected cell models, HBV transcription is mainly derived from cccDNA. This distinction may lead to different results. Interestingly, Saso et al. (2018) applied the AlphaScreen technology and reported that rapamycin could inhibit the entry of HBV into cells and that this is not associated with the inhibition of mTOR but instead appears to be due to its direct interaction with NTCP. However, in this study, we used the cre-rcccDNA model in Huh-7 cells and found that rapamycin also plays an important role in processes other than cell invasion, including HBx suppression and cccDNA transcription, and these effects were largely related to mTOR. Besides, it was reported that HBx played a greater role in transcriptional control in PHHs than in HepG2-NTCP cells (Lucifora et al., 2011). However, in our study, the inhibitory effect of rapamycin on cccDNA transcription in HepG2-NTCP and PHH cells was similar (approximately 40–60%). This phenomenon may be caused by a variety of reasons. First, different sources and qualities of different PHHs and HepG2-NTCP cells may have different efficiencies for HBV infection. Second and more importantly, PHH cells serve as a cell model that can support long-term infection and replication of the HBV virus, and most of the experiments using this model treated cells for a long time (more than 2 weeks). However, in our experiments, due to the limitations of the cells and culture conditions, we only added drugs and treated the cells for 6 days, and the entire culture process did not exceed 10 days from the beginning of seeding. Importantly, the effect of HBx on cccDNA transcription showed a greater function over time, which is probably related to the important role of HBx in the maintenance and stability of cccDNA (Levrero et al., 2009; Lucifora et al., 2011; Decorsiere et al., 2016). Notably, since rapamycin only reduced the protein level of HBx without eliminating HBx protein, the experimental results may be different from those of HBx knockout experiments.

Overall, in this study, we found that rapamycin inhibits HBx protein expression. This small-molecule rapamycin shows an effective anti-HBV activity, especially against the transcription of cccDNA, by promoting HBx degradation mediated by the UPS. These results showed that rapamycin could serve as a potential candidate for the functional cure of CHB. However, as the HBV genome integrated into the host genome is not derived from cccDNA transcription, rapamycin has limited effectiveness on integrated HBV DNA expression. In addition, further research is needed on the duration of rapamycin treatment required for anti-HBV treatment and its toxicity and side effects. Moreover, whether HBx may rebound after cessation of treatment and how to develop better combination treatments needs additional experimental evidence.

## Data availability statement

The original contributions presented in the study are included in the article/Supplementary material, further inquiries can be directed to the corresponding authors.

## Ethics statement

The animal study was reviewed and approved by the Animal Ethics Committee of Chongqing Medical University.

## Author contributions

Z-ZZ and J-HR conducted the experimental design. YZ, JC, and JL drafted the manuscript. YZ and LL performed most of the experiments. YZ and S-TC analyzed the experimental data. Y-PQ, XH, and D-QW helped to perform the animal experiments. FR, FL, and H-BY contributed to conducting northern blotting and southern blotting analysis. All authors revised the manuscript critically and approved the final manuscript.
