# Supplementary material for: Rapamycin inhibits hepatitis B virus covalently closed circular DNA transcription by enhancing the ubiquitination of HBx
Source: Front Microbiol. 2022 Aug 11;13:850087. doi: 10.3389/fmicb.2022.850087 (PMC9403416; doi:10.3389/fmicb.2022.850087)
Supplement: Supplementary file 2 [file Table_1.DOCX]

**Supporting Information**

**Supplementary Figure legends**

**Figure S1. Alamar blue assay of different cells after treating with rapamycin.** Huh-7 cells were treated with the indicated concentrations of rapamycin for 3 days, HepG2-NTCP and PHH cells were treated with the indicated concentrations of rapamycin for 6 days. Then the cells were carried out alamar blue assay and the medium was collected for detecting. The data were presented as mean ± SD of three independent experiments.

**Figure S2. Rapamycin did not affect the stability of HBc, HBs and Pol protein.** Huh-7 cells transfected with 3×Flag-HBc, 3×Flag-HBs or 3×Flag-Pol plasmid were exposed to 5 μM rapamycin and 10 μg/ml cycloheximide at the indicated times. The levels of the proteins were examined by western blotting analysis, and GAPDH was used as the loading control.

**Figure S3. Ubiquitin proteasome inhibitor MG132 eliminated the inhibiting effect of rapamycin on endogenous HBx**. HBV-infected HepG2-NTCP cells were exposed to 5 μM rapamycin for 6 days, and MG132 was added 8 h before the cells were harvested. The levels of the proteins were examined by western blotting analysis, and GAPDH was used as the loading control.

**Figure S4. Rapamycin blocked cccDNA transcription in HBV-infected PHH cells.** PHH cells were infected with HBV particles in the presence of 4% PEG8000 for 24 h. Then HBV-infected PHH cells were exposed to indicate concentration of rapamycin for 6 days. **(A)** The level of total HBV RNAs and 3.5-kb RNA were detected by real-time PCR using specific primers. β-actin mRNA level was used as an internal control. **(B)** The level of HBV core DNA was analyzed by real-time PCR. **(C)** The levels of secreted HBsAg and HBeAg in cell culture supernatant were detected by ELISA. **(D-E)** The level of cccDNA was analyzed by real-time PCR and the ratios of HBV 3.5-kb RNA/cccDNA, total RNAs/cccDNA were calculated. The data were presented as mean ± SD of three independent experiments. ***, *P* < 0.05

**Figure S5. Rapamycin blocked HBV (r)cccDNA transcription in the (r)cccDNA cell model.** Huh-7 cells were co-transfected with precursor rcccDNA and pCMV-KRAB-Cre plasmids. Cells were harvested after rapamycin treatment at indicated concentrations for 4 days. **(A)** The level of total HBV RNAs and 3.5-kb RNA were detected by real-time PCR. β-actin mRNA level was served as an internal control. **(B)** HBV core DNA was analyzed by real-time PCR. **(C)** Secreted HBsAg and HBeAg in cell culture supernatant were detected by ELISA. **(D)** The levels of HBx, HBc and HBs protein were detected by western blotting analysis and GAPDH was used as the loading control. The data were presented as mean ± SD of three independent experiments. ***, *P* < 0.05.

**Figure S6. Knockdown of mTOR increased the ubiquitination level of HBx protein.** Huh-7 cells were transfected with 3×Flag-HBx and UBB plasmids and were then transfected with siRNAs for mTOR. Then the cells were treated with 5 μM rapamycin for 24 h. The ubiquitination level of HBx protein in Huh-7 cells were examined by Co-IP and western blotting analysis, GAPDH served as the loading control.***, *P* < 0.05.

**Supplementary Table**

| total HBV RNAs forward | 5’- ACCGACCTTGAGGCATACTT-3’ |
| --- | --- |
| total HBV RNAs reverse | 5’- GCCTACAGCCTCCTAGTACA-3’ |
| HBV 3.5-kb RNA forward | 5’- GCCTTAGAGTCTCCTGAGCA-3’ |
| HBV 3.5-kb RNA reverse | 5’- GAGGGAGTTCTTCTTCTAGG-3’ |
| β-actin mRNA forward | 5’-CTCTTCCAGCCTTCCTTCCT-3’ |
| β-actin mRNA reverse | 5’- AGCACTGTGTTGGCGTACAG-3’ |
| HBV cccDNA forward | 5’- GTGCACTTCGCTTCACCTCT-3’ |
| HBV cccDNA reverse | 5’-AGCTTGGAGGCTTGAACAGT-3’ |
| HBV cccDNA probe | 5’-TTCATCCTGCTGCTATGCCTGATCTTCTTG-3’ |
| HBV core DNA forward | 5’-CCTAGTAGTCAGTTATGTCAAC-3’ |
| HBV core DNA reverse | 5’-TCTATAAGCTGGAGGAGTGCGA-3’ |
| si-mTOR1 forward | 5’-CUGUCAGAAUCCAAGUCAATT-3’ |
| si-mTOR1 reverse | 5’-UUGACUUGGAUUCUGACAGTT-3’ |
| si-mTOR2 forward | 5’-GCAUGGAAGAAUACACCUGUA-3’ |
| si-mTOR2 reverse | 5’-UACAGGUGUAUUCUUCCAUGC-3’ |
| HBx K95R forward | 5’-AAATATTGCCCAAGGTCTTACATCGGAGGACTCTTGGACTCTCA  GCAATG-3’ |
| HBx K95R reverse | 5’-CATTGCTGAGAGTCCAAGAGTCCTCCGATGTAAGACCTTGGGCA  ATATTT-3’ |
