# Supplementary figures and images for: Rapamycin inhibits hepatitis B virus covalently closed circular DNA transcription by enhancing the ubiquitination of HBx
Source: Front Microbiol. 2022 Aug 11;13:850087. doi: 10.3389/fmicb.2022.850087 (PMC9403416; doi:10.3389/fmicb.2022.850087)

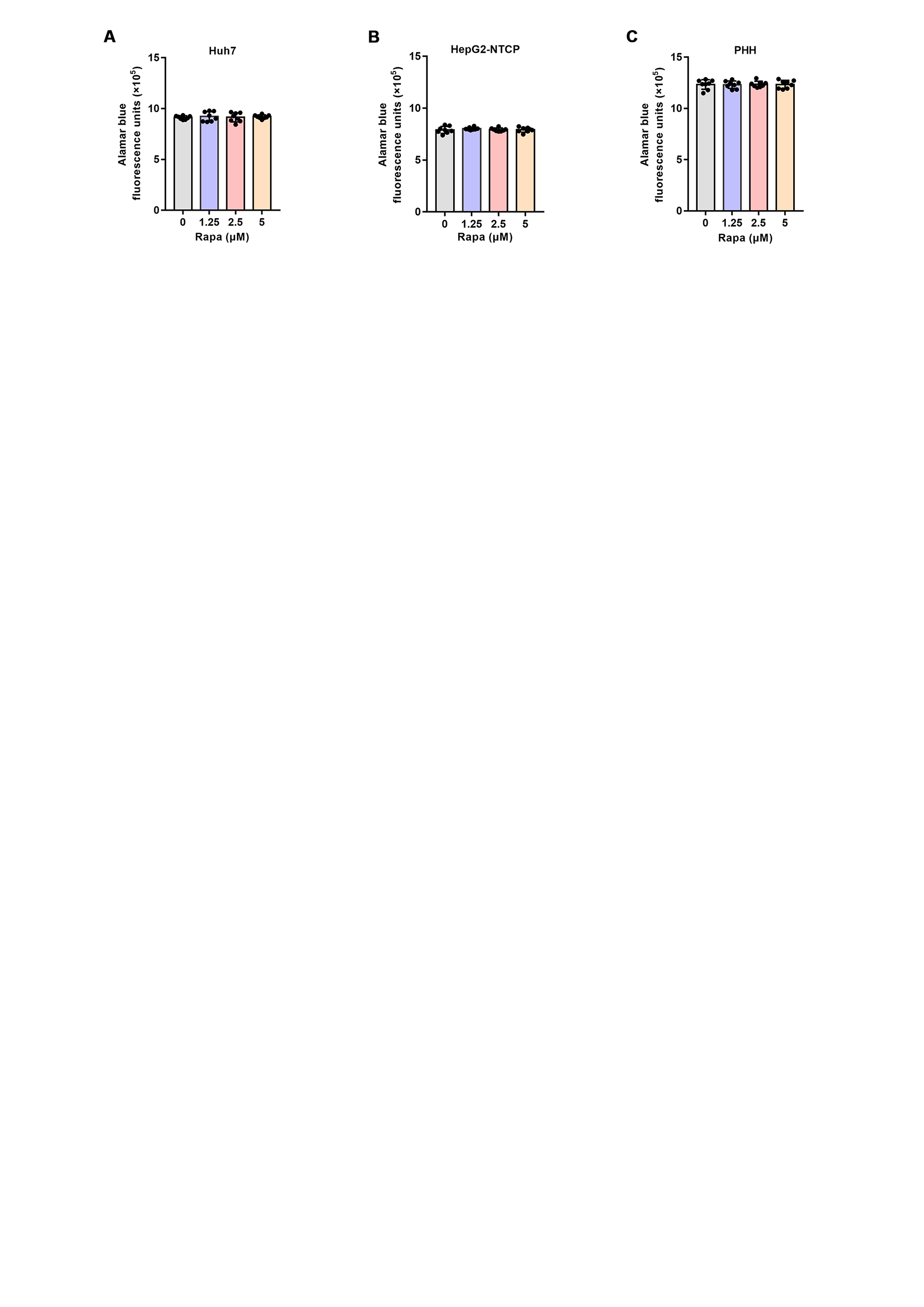

Supplement: Supplementary file 3 [file Image_1.TIF]

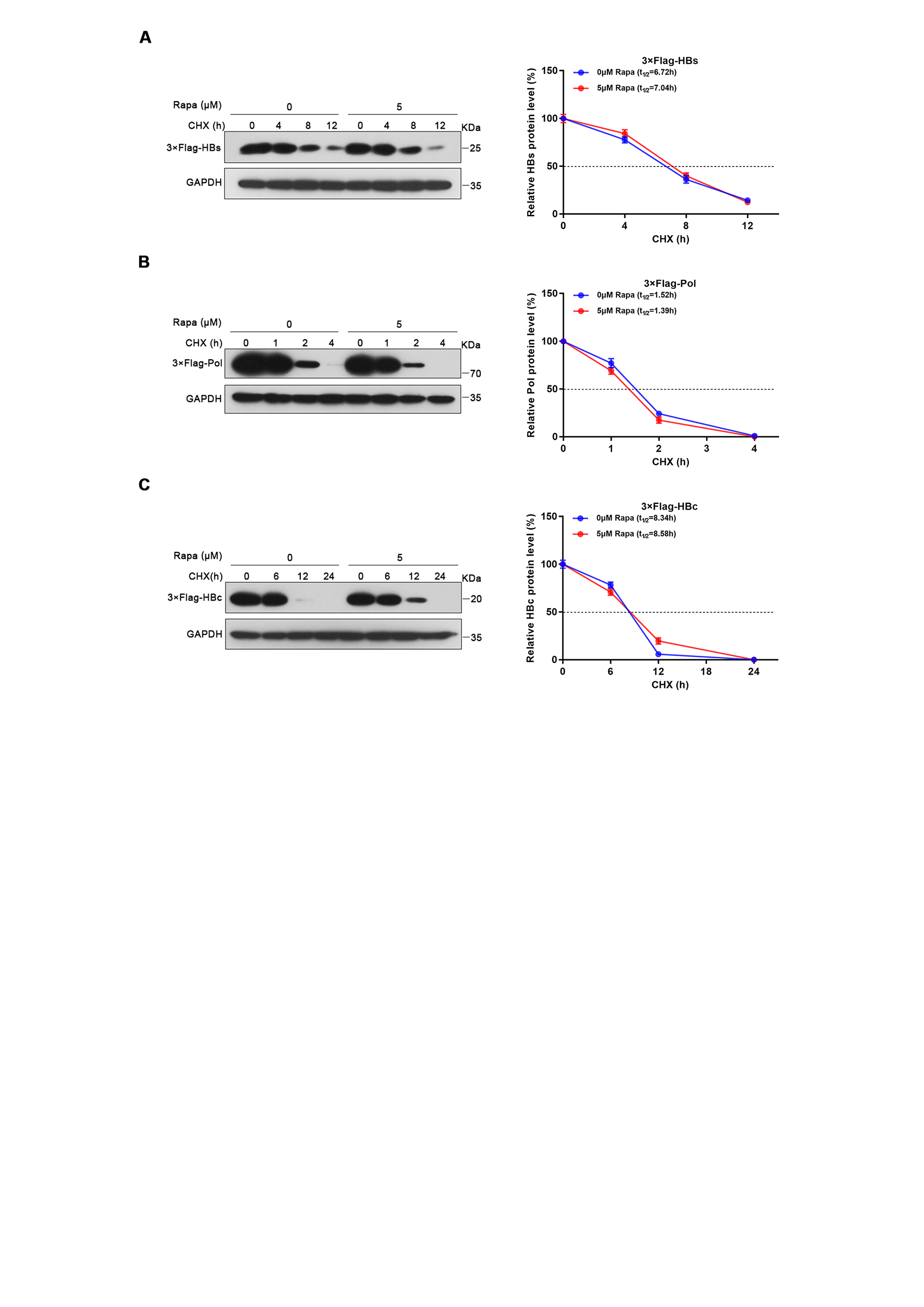

Supplement: Supplementary file 4 [file Image_2.TIF]

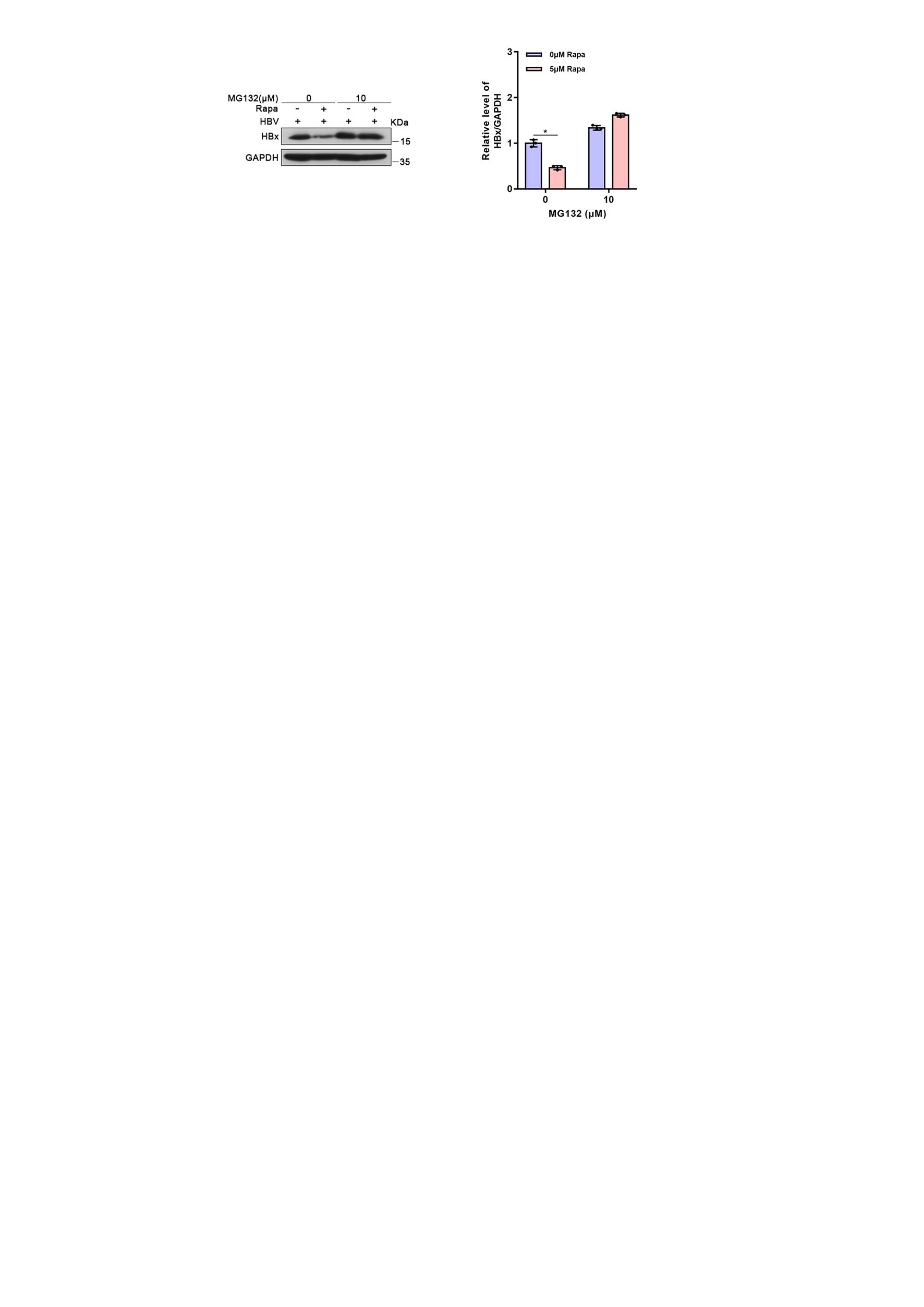

Supplement: Supplementary file 5 [file Image_3.TIF]

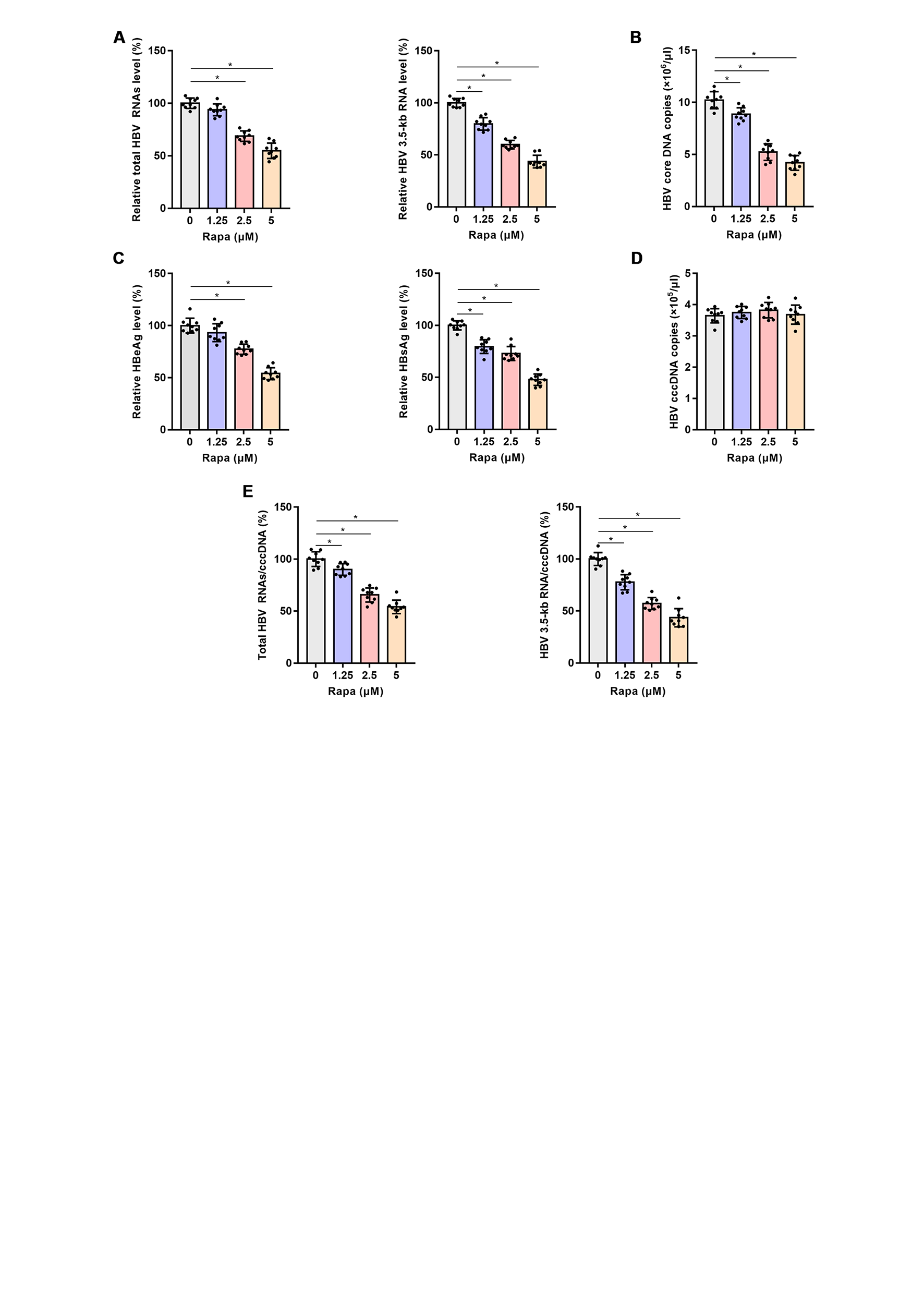

Supplement: Supplementary file 6 [file Image_4.TIF]

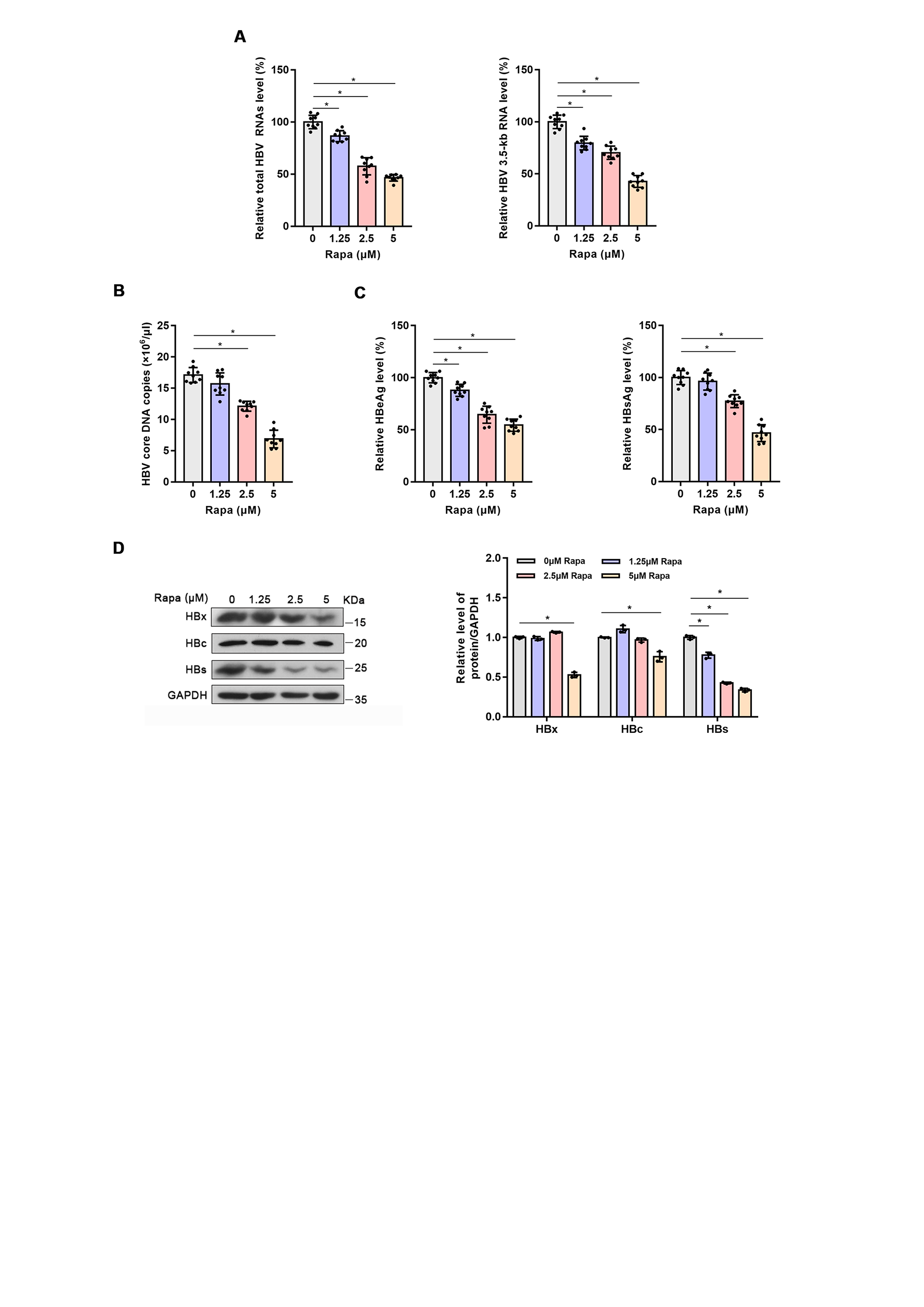

Supplement: Supplementary file 7 [file Image_5.TIF]

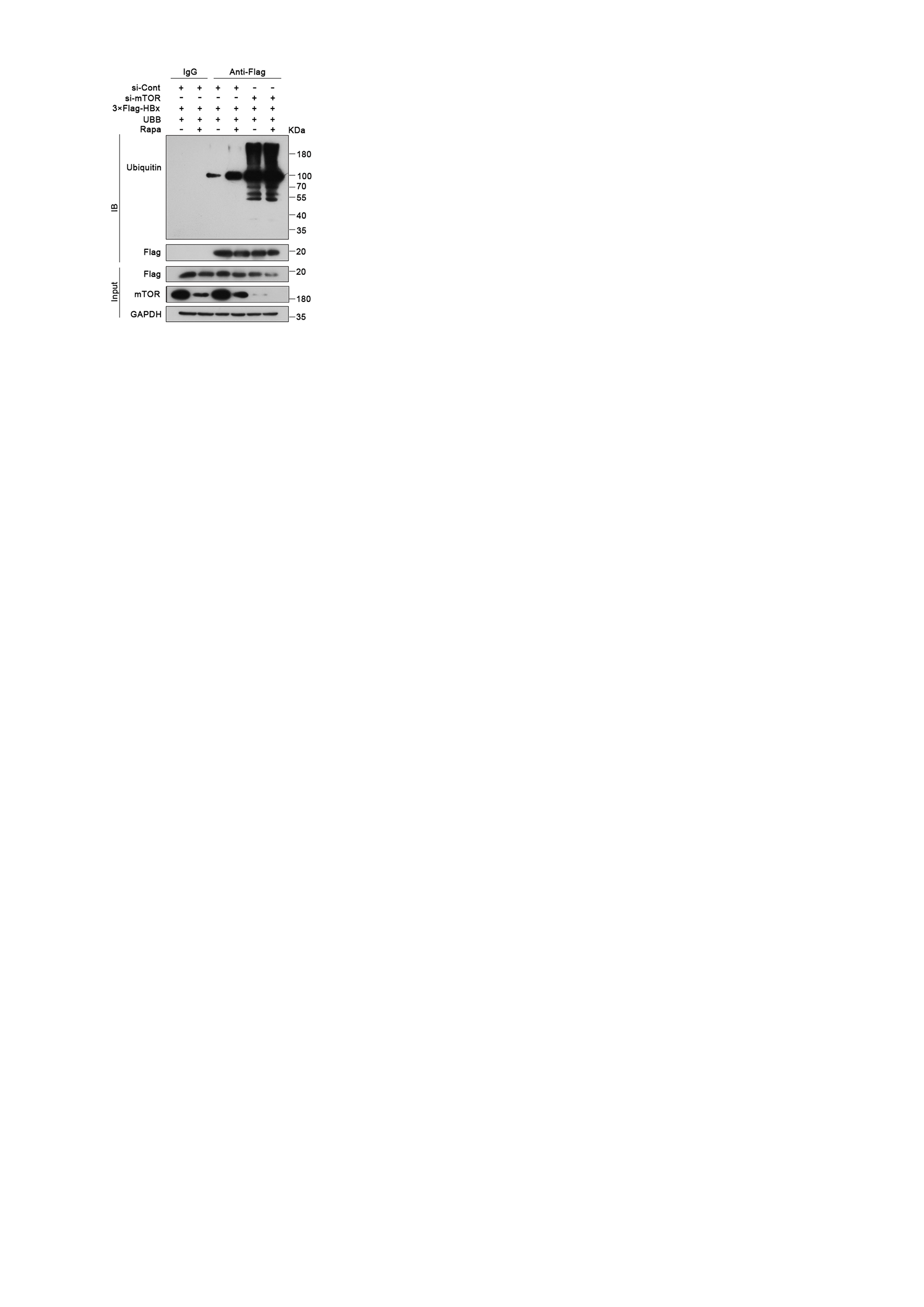

Supplement: Supplementary file 8 [file Image_6.TIF]
